# Mentoring the Mentors: Implementation and Evaluation of Four Fogarty-Sponsored Mentoring Training Workshops in Low-and Middle-Income Countries

**DOI:** 10.4269/ajtmh.18-0559

**Published:** 2018-11-14

**Authors:** Monica Gandhi, Tony Raj, Ryan Fernandez, Laetitia Rispel, Nonhlanhla Nxumalo, Andrés G. Lescano, Elizabeth A. Bukusi, Blandina T. Mmbaga, Douglas C. Heimburger, Craig R. Cohen

**Affiliations:** 1 Department of Medicine, University of California, San Francisco (UCSF), San Francisco, California;; 2 St. John’s Research Institute (SJRI), Bangalore, India;; 3 Faculty of Health Sciences, Centre for Health Policy, School of Public Health, University of the Witwatersrand, Johannesburg, South Africa;; 4 Emerge, Emerging Diseases and Climate Change Research Unit, School and Public Health Administration, Universidad Peruana Cayetano Heredia, Lima, Peru;; ^5^Centre for Microbiology Research, Kenya Medical Research Institute (KEMRI), Nairobi, Kenya;; 6 Kilimanjaro Clinical Research Institute (KCRI) and Kilimanjaro Christian Medical University College (KCMUCo), Moshi, Tanzania;; 7 Vanderbilt Institute of Global Health, Nashville, Tennessee;; 8 Department of Obstetrics, Gynecology, and Reproductive Sciences, University of California, San Francisco (UCSF), San Francisco, California;; 9 University of California Global Health Institute, San Francisco, California

## Abstract

A growing body of evidence highlights the importance of competent mentoring in academic research. We describe the development, implementation, and evaluation of four regional 2-day intensive workshops to train mid- and senior-level investigators conducting public health, clinical, and basic science research across multiple academic institutions in low- and middle-income countries (LMICs) on tools and techniques of effective mentoring. Sponsored by the Fogarty International Center, workshops included didactic presentations, interactive discussions, and small-group problem-based learning and were conducted in Lima, Peru; Mombasa, Kenya; Bangalore, India; and Johannesburg, South Africa, from 2013 to 2016. Mid- or senior-level faculty from multiple academic institutions within each region applied and were selected. Thirty faculty from 12 South America–based institutions, 29 faculty from eight East Africa–based institutions, 37 faculty from 14 South Asia–based institutions, and 36 faculty from 13 Africa-based institutions participated, with diverse representation across disciplines, gender, and academic rank. Discussions and evaluations revealed important comparisons and contrasts in the practice of mentoring, and specific barriers and facilitators to mentoring within each cultural and regional context. Specific regional issues related to hierarchy, the post-colonial legacy, and diversity arose as challenges to mentoring in different parts of the world. Common barriers included a lack of a culture of mentoring, time constraints, lack of formal training, and a lack of recognition for mentoring. These workshops provided valuable training, were among the first of their kind, were well-attended, rated highly, and provided concepts and a structure for the development and strengthening of formal mentoring programs across LMIC institutions.

## Introduction

The importance of effective mentorship in the development, success, and retention of trainees and early career investigators in academic research settings is increasingly recognized.^1–11^ For example, early career faculty in institutions with strong mentoring programs demonstrate greater research productivity than faculty in institutions without such programs.^2^ Standardized mentoring support programs can triple the success of junior faculty in terms of typical metrics (e.g., grants and articles) of academic performance.^11^ Successful mentoring programs, however, require skilled mentors. Despite a burgeoning understanding that faculty can benefit from mentoring training,^12,13^ there are only a limited number of formal mentorship training programs^14^ for academic researchers in low- and middle-income country (LMIC) settings that offer faculty the opportunity to develop skills and incorporate best practices.

As there is good evidence that trained mentors, as opposed to untrained mentors, are more effective in their interaction with, and support of their mentees,^13^ inadequate mentoring may be a “modifiable risk factor” in the disparate patterns of academic progress observed for early stage investigators in LMIC settings. Robust mentorship training programs geared specifically toward training mentors in the skills and practices that cultivate effective mentoring relationships with early stage investigators could improve the depth and success of the LMIC global health academic workforce. However, there is a dearth of empirical data on the relative efficacy of mentor training methods. Although graduate and health profession programs often provide some content related to overall teaching skills, most graduates are expected to perform mentoring activities eventually without any formal training in the area. These mentors often perform the role *ad hoc* or may mimic (intentionally or not) mentors they have interacted with in their own careers, with mixed results in terms of effectiveness and mentee-reported satisfaction with the mentoring relationship.^15–17^ Experiential and/or practical mentoring approaches may improve mentorship skills, especially in resource-limited settings. However, theory-based programs designed to provide systematic training in mentoring and reinforce standard approaches that can be tailored to individual needs, although limited, have been highly effective.^13,14,18^

The purpose of this article is to describe the development, implementation, and evaluation of a mentorship workshop to train mid- and senior-level investigators conducting public health, clinical, and basic science research across multiple academic institutions in LMICs to be more effective mentors. The programs were sponsored by the six-funded Fogarty International Center Global Health Program for Fellows and Scholars and were developed collaboratively between United States–based investigators with expertise in mentoring^3–5^ and senior faculty and academic leaders at academic institutions in Latin America, East Africa, South Asia, and Southern Africa. Investigators from multiple academic institutions in regions proximal to the hosting academic institution applied and were accepted to the workshops. This article describes the results of each workshop on the practices and facilitators of, and barriers to, mentoring; action plans by individual participants; evaluations and follow-up actions to establish local mentoring programs in each region; and post-workshop surveys to evaluate the impact of the trainings on local mentorship practices and programs.

## Methods

### Background and organizers of the “mentoring the mentors” training programs.

#### Role of Fogarty International Center.

The Fogarty International Center at the National Institutes of Health (NIH) supports six United States—based university consortia to provide mentored global health research training opportunities in LMICs through the Global Health Program for Fellows and Scholars. The six consortia are focused at Harvard University, Cambridge, MA; University of California Global Health Institute (UCGHI) based at the University of California, San Francisco (UCSF), the University of California, Los Angeles, the University of California, San Diego, and the University of California, Davis; University of North Carolina, Chapel Hill; University of California, Berkeley; University of Washington, Seattle, WA; and Vanderbilt University, Memphis, TN. The GloCal Health Fellowship Consortium at UCGHI, which supports fellows from all 10 University of California campuses, initiated the “Mentoring the Mentors” training workshops in 2013 to train faculty across all international sites supported by the Global Health Program. All six of the consortia contributed resources and faculty to one or more of the four “Mentoring the Mentors” workshops held in Lima, Peru, in May 2013; Mombasa, Kenya, in June 2013; Bangalore, India, in November 2014; and Johannesburg, South Africa, in March 2016.

#### Role of the LMIC university faculty and leadership.

Each “Mentoring the Mentors” workshop in the four LMICs hosted faculty participants from a number of neighboring academic institutions within the region. Each program was developed collaboratively between local faculty and faculty leadership at the host institution and neighboring institutions, with input from UCSF-based faculty on standard program content. Each training program was designed to cover overarching principles of mentorship through a series of didactic trainings, along with a number of small group sessions and large group discussions focusing on the cultural context of mentoring, along with specific barriers and facilitators of mentoring, within each region.

#### Role of GloCal and UCSF.

One impetus of the GloCal Health Fellowship Consortium and faculty based at UCSF to help lead this initiative stemmed from strong mentoring initiatives and “Mentoring the Mentor” training programs already being conducted at UCSF through other NIH-funded initiatives.^3–5,12,19–21^ For instance, the UCSF Center for AIDS Research had established a strong mentoring program in the years before the development of this initiative which had already launched initiatives to mentor early career investigators in HIV research^21^ and provide mentoring training programs for HIV researchers across the United States.^3–5,20^ In addition, the UCSF-based Clinical and Translational Science Institute (CTSI) launched a robust Mentor Development Program (MDP) in 2006,^19^ where faculty receive intensive training over a year in mentorship techniques and are provided with tools to enhance mentee success. This program helped define a set of core competencies for mentors in clinical and translational research.^22^ An interim evaluation of the MDP program demonstrated a sustained impact on mentoring skills, techniques, and focus.^12^

#### Faculty participants in each program.

Each training program was developed iteratively over 6–8 months via conference calls between UCSF-based faculty, faculty based at other Global Health Program consortia in the United States, and faculty leadership at the host and neighboring international institutions. The LMIC leadership included deans or department chairs of the hosting institutions, along with Division/Section Heads and key investigators involved in mentoring initiatives. A call for applicants from institutions in LMICs associated with each consortia was made approximately 3 months before each workshop and applicants submitted a curriculum vitae and a paragraph summarizing their interest in mentoring training. Each regional workshop was restricted to around 30 participants to keep the format intimate and interactive, with preference given to mid-level and senior faculty at institutions from neighboring countries who were currently mentoring junior investigators and were in positions to help develop mentoring capacity at the institutional level.

### Format of the training programs.

Each 2-day workshop included formal presentations, interactive discussions, and small-group problem-based learning activities. Because each workshop had been developed iteratively over time with intimate participation by local faculty leaders, each workshop was unique in its tailored content, although some core principles of mentoring were covered uniformly. Table 1 summarizes the general topics covered in each workshop.

**Table 1:** Training areas and specific topics covered in each “mentoring the mentors” workshop (standard content intermingled with locally developed and tailored content)

Training area	Specific topics	Description
Emotional intelligence	Knowing your own and others’ personality styles and how to work together	Working with mentees with different interpersonal and work styles.
	Mindfulness	Being fully aware, present, and non-distracted when interacting with mentees
Communication	Giving and Receiving Feedback	How to give mentees feedback in a constructive way.
		How to solicit feedback on your mentoring.
	Dealing with professional interpersonal conflict	Recognizing and communicating effectively and early when problems are emerging
	Setting Expectations	Setting clear expectations in the mentoring relationship and providing the framework for a “mentee-driven relationship”
		Defining mentoring team members’ roles
	Distance mentoring	Distance mentoring—tools and techniques, and pitfalls and advantages
Professional skills	Time Management	How to make time for mentoring and use it effectively
		How to teach time management skills to mentees
	Individual Development Plans and Mentoring Tools/Resources	Developing and making the most of IDPs for mentees.
		Developing and implementing one’s own mentoring IDP to set and monitor goals related to mentoring skills
		Navigating the mentoring tools and resources from the UCSF CTSI
	Life–Work Balance	Understanding your own life-work balance and examples from others in academic research.
		How to support life–work balance in your mentees
	Team Science	Working effectively as part of a mentoring team
		Teaching mentees how to work as part of a research team and team leadership
	Negotiation	Teaching your mentee negotiation skills.
		Negotiating protected time and acknowledgement for mentoring
		Professional ethics
Diversity	Microaggressions	Identifying and reducing microaggressions in the mentoring relationship/institution
	Unconscious Bias	Awareness of one’s own unconscious bias and how it might affect mentoring relationships and effectiveness
		Celebrating diversity and recognizing social constructs that define differences
		Recognize internalized superiority and internalized inferiority
		Developing critical diversity literacy

CTSI = clinical and translational science institute; IDP = individual development plan; UCSF = University of California, San Francisco.

The workshops included skill-based learning in a number of topics including: setting goals and expectations for the mentor–mentee relationship; developing a mentoring philosophy; enhancing communication strategies, and identifying and resolving challenges; time management strategies for mentors and mentees; navigation of work-life balance in a research career; sharing online tools and resources developed by the UCSF CTSI’s Mentoring Program; strategies to enhance institutional commitment to mentoring programs; how to mentor mentees in grant writing and manuscript preparation; mentor and mentee evaluation and bidirectional feedback strategies; barriers and facilitators to mentoring at the global and local level; addressing diversity in the mentor–mentee relationship and challenges for mentees from diverse backgrounds in each setting (e.g., Latin America, Africa, and South Asia) in academic institutions; and developing a framework and action plan for launching or strengthening mentoring programs at each represented institution. Each program was launched by a keynote speech, usually from a recognized senior leader from the hosting institution in each region.

Because of the importance of peer-to-peer mentoring support, each program ended with a “Mentor Consultation Clinic,” which involved organization of the participants into small groups (five to seven mentors each). Each small group was presented with a strict structure for the “clinic” in which one person presented a mentoring challenge (2–3 minutes), followed by an information-gathering/question period from the group (5 minutes), ultimately leading to recommendations from the group for addressing the challenge (5 minutes). Mentors in each clinic were provided with each other’s contact information and groups were encouraged to continue informal peer-to-peer mentoring relationships after each program had ended.

### Data from each training program.

**Figure 1.**
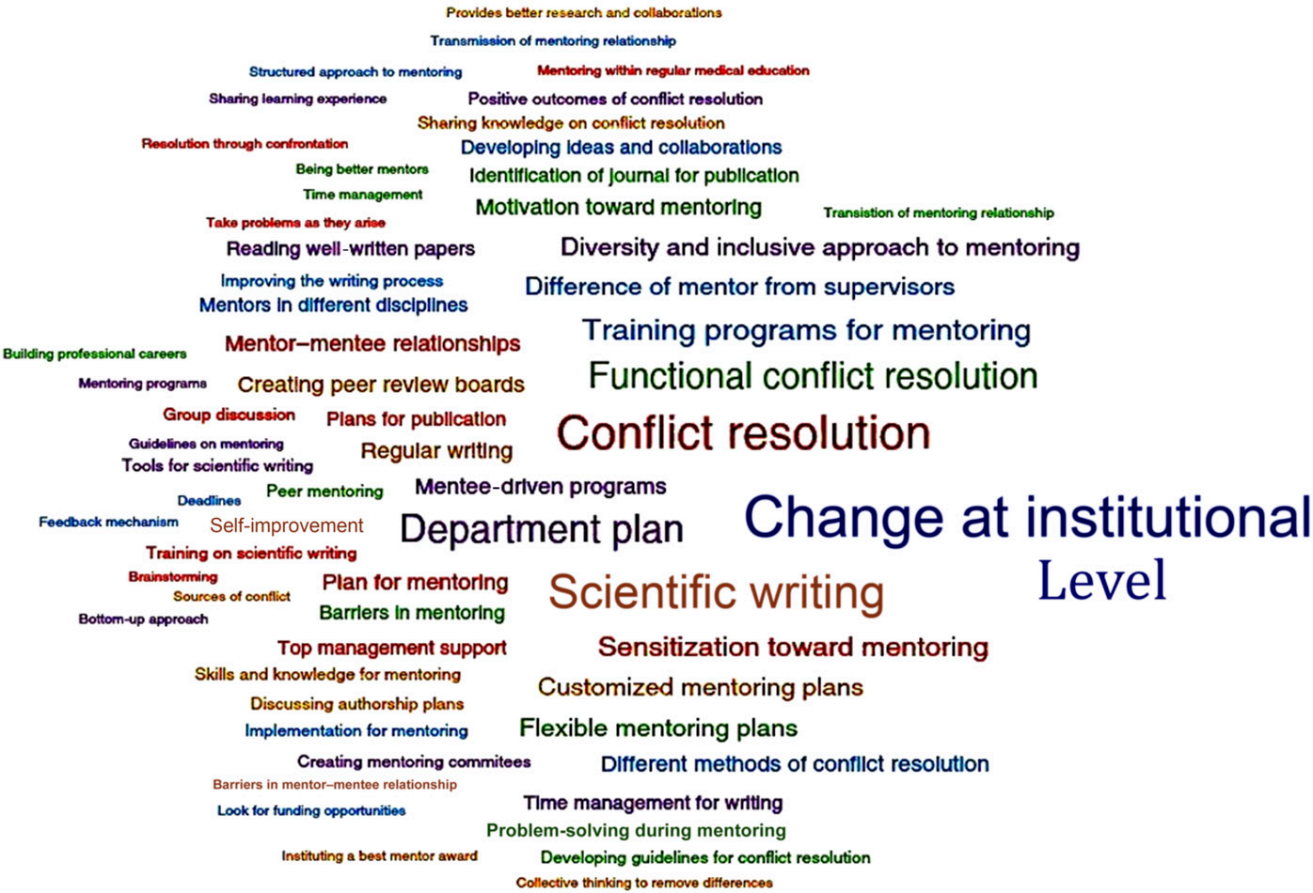
Major themes that emerged from the mentor action plans from the South Asia workshop, Bangalore, India 2014. A thematic analysis (provided by Dedoose Version 8.0.35 [2018]; SocioCultural Research Consultants, LLC) on the main themes emerging from the mentoring action plans for the mentoring workshops described in this article. The size of the text in the “wordcloud” indicates the frequency of the theme expressed in the Mentor Action Plans (so that “change at the institutional level” indicates the most frequently cited theme to emerge from the participants to move mentoring forward globally). The blue text indicates changes that need to be instituted at the institutional and departmental levels; the green text indicates changes that need to occur at the mentor level; and the red text indicates changes that need a dialog between mentor and mentee. This figure appears in color at www.ajtmh.org.

Before each workshop, a pre-meeting survey was sent out to assess the academic rank of each participant; experience in mentoring; and current number and training level of mentees. Additional data provided by the workshops came from the small group activities in each workshop where participants identified barriers to mentoring at the individual, institutional, and global levels and proposed possible solutions. Finally, a Mentor Action Plan (MAP) was completed by each participant at the end of the workshop. The MAP solicited each participant’s self-reported individual strengths and areas of improvements as a mentor, learning points from that workshop’s activities, and action plans for implementing recommendations and techniques covered in the workshop in their individual mentoring activities going forward. Copies of the MAPs were retained for qualitative analysis. Members of the project team who were independent of those who designed and implemented the program conducted thematic analysis of the MAPs as presented in Results. The thematic analysis presented in Figure 1 was generated by Dedoose Version 8.0.35 (2018); SocioCultural Research Consultants, LLC, Los Angeles, CA.

### Technical advisory meeting at conclusion of four training programs.

During the fifth year of the program, GloCal hosted a senior-level technical advisory meeting on global health mentoring at LMIC institutions before the Consortium of Universities for Global Health conference in April 2017. Low- and middle-income countries leaders of the four “Mentoring the Mentors” workshops, along with the principal investigators from each of the six Fogarty International Center Global Health Programs for Fellows and Scholars, along with staff from the NIH FIC, gathered to discuss and plan how the program can continue to best support the development, sustainability, and productivity of strong mentorship programs for global health researchers across LMIC institutions. Before this advisory meeting, a survey was sent out to participants in all four “Mentoring the Mentors” training programs to elicit further feedback on the content of the training programs and assess the impact of the training programs on subsequent mentoring efforts and establishment of mentoring programs at participating LMIC institutions.

## Results

### Participant composition.

Thirty faculty from 12 Latin America–based institutions participated in the 2013 workshop held in Lima, Peru; 29 faculty from eight East Africa–based institutions participated in the 2013 workshop held in Mombasa, Kenya; 37 faculty from 14 South Asia–based institutions participated in the 2014 workshop held in Bangalore, India; and 36 faculty from 13 Africa-based institutions participated in the 2016 workshop held in Johannesburg, South Africa. Of the 132 mentors trained in the four workshops from 2013 to 2016, 29% were at the full-professor level at their institutions; 21% were associate professors; 18% were assistant professors; 32% were instructors or in another academic series; and 24% of participants were section heads, department chairs, or held other major leadership positions at their respective institutions. Participants of the four workshops were 55% female and each participant had a median of four primary mentees (range 0–12 mentees).

### Shared and unique barriers and facilitators to mentoring identified at workshops.

Table 2 summarizes the common barriers and proposed solutions to mentoring effectively in each region where the workshops were conducted.

**Table 2:** Common individual and institutional/global barriers to mentoring identified at the workshops with proposed solutions

Barrier	Proposed solution
**Institutional/global barriers**	**Solutions**
Resource constraints	Urge governments to recognize the value of mentoring to the biomedical or global health research workforce and support institutions to support mentoring
No resources to put into practice a true cultivation of mentees	
Lack of resources in information-communication technology which could aid in mentoring efforts, including distance mentoring	
Sheer numbers of trainees compared with mentors	
	Ensure that each grant put in by individuals at an institution includes training and development funds for mentees
Lack of infrastructure e.g. private office space	
Lack of mainstreaming of mentoring in many institutions	
Mentoring not part of the institutional culture or framework	
	Develop an institutional framework with documentation for formalizing mentoring
	Approach deans or other institutional leaders to propose a structured mentoring the mentors training program at each institution, and a mentoring program or “mentoring office” that provides resources and support to both mentees and mentors. Develop guidelines for the institution to regulate and track mentoring (e.g. how many mentees each mentor can have, etc.)
No recognition at the level of the institution for mentoring (neither recognized nor encouraged)	
Few or no opportunities within an institution to implement new or innovative programmes, including mentoring programs	
Resistance to change within institutions	
Lack of awareness of the need for mentoring	
Lack of guidelines within institutions on how to mentor and how to structure the mentor–mentee relationships	
Lack of nomenclature to define a “mentor” in LMIC institutions e.g. what is a mentor compared with an advisor	
No training opportunities for mentors	
Inequalities in support for mentoring local mentees vs. mentees who come from high-income countries/institutions (which will impact mentors’ ability to be present for local mentees)	
Lack of opportunities for networking between mentors	
	Make a business case of the benefits to the institution of effective mentoring e.g. more talented faculty coming up in ranks.
	Define roles and nomenclature of mentoring so that institutions can start incorporating mentoring language
	Provide protected time for mentoring to faculty members
	Develop incentives for faculty to mentor and to mentor effectively e.g. recognition, mentoring awards, credit toward promotion
	Make existing mentoring tools widely available and accessible to mentors and mentees across the institution
	Initiate a global initiative, an Academy of Mentors globally
	Establish small local mentoring groups for peer-to-peer support in mentoring

**Individual-level barriers**	**Solutions**
Lack of funding for mentoring	Training programs for mentors on mentoring skills
Lack of time or competing priorities	
Mentor having bad experiences or no experience with mentorship (never been mentored)	
	Time-management skills training for both mentors and mentees
Unclear benefit to the mentor	
Lack of or little experience of the mentor in mentoring	
	Formalize an orientation program for incoming students to the mentorship culture of institution
Intimidating mentors	
Lack of insight into mentee’s experience or assets	
Mentee not taking initiative	
Perception of threat or competition (real or perceived) from the mentee	Creating a local mentoring training program at each institution
	Clearly define expectations of a mentor–mentee relationship through a written record
Unrealistic expectations of mentees	
Poor communication skills of both mentor and mentee	
	Allow multiple levels of communication between mentor and mentee; allow for true feedback
Personality differences between mentor and mentee	Implementing policies that provide structure to the mentor–mentee relationship
	Individual changes in mentoring practices to structure the mentor–mentee meetings and establish regularity, initiate and maintain biannual IDPs to keep a written record of expectations, and provide opportunities for bidirectional feedback in the mentoring relationship
	Realizing that mentor–mentee relationships should be truly reciprocal and can reduce the resentment toward mentoring without institutional recognition
Cultural differences in the relationship	
Culture that does not encourage dialog—mentee cannot “talk back”	
Lack of honesty and straightforwardness in the mentor–mentee relationship	
Hierarchy (power dynamics)	
Gender dynamics	

IDP = individual development plan; LMIC = low- and middle-income country.

Barriers identified for effective mentoring by various faculty independent of region (Table 2) were a lack of an institutional mentoring culture, institutional failure to acknowledge or “give credit” for mentoring activities in the merit or promotions process, a general lack of time or time-management strategies to balance mentoring with other academic pursuits, and a lack of support for mentoring and its challenges. Other barriers identified were the lack of an organized structure in mentoring relationships (including frequency of meetings, documentation of expectations, and goal monitoring), unclear expectations of a mentor–mentee relationship, discomfort with difficult conversations, and a lack of training on how to provide feedback. Some cited a lack of institutional structure to the mentoring process as a barrier to effective mentoring, with mentor–mentee relationships being created in an ad hoc manner without institutional oversight or attention to balancing the number of mentees per mentor in part because of a perceived lack of well-qualified mentors.

A variety of solutions, applicable to different regional contexts to different degrees, solu were proposed to improve the effectiveness of the mentor–mentee relationship. These included the implementation of a formalized structure of the mentoring process as developed by the UCSF CTSI program and taught in each workshop. Faculty participants stated that they planned to establish a regular meeting schedule with their mentees and implement individual development plans (IDPs) to be filled out at least annually by mentees to identify expectations and goals, and monitor progress and troubleshoot impasses. Faculty planned to provide greater clarity in delineating expectations for their mentees, including establishment of meeting agendas and follow-up procedures following regular meetings. Many faculty planned to approach their division chiefs, department chairs, or deans to suggest mentoring training programs such as this workshop at their institutions and to aid in the establishment of a greater culture of mentoring, including funding for mentoring efforts and adding mentoring to the criteria list for academic rank promotion. Participants all cited a need for more formalized mentoring workshops such as those provided by these workshops at their institutions, and formal mentoring programs to both structure and evaluate the progress of mentoring relationships over time.

Table 3 summarizes unique challenges to mentoring that were specific to the particular region or set of institutions represented in each workshop. Data for these tables were transcribed from notes taken during the report-back sessions on the individual and institutional/global barriers to mentoring conducted on the first day of each workshop. Data from the individual Mentoring Action Plans and the discussions of diversity in each cultural context, including direct quotes, also contributed to the data presented in Table 3.

**Table 3:** Challenges or facilitators of mentoring that were unique to each region

Workshop	Unique issues
Lima, Peru, Workshop May 2013	Expectations of funding agencies and research community to conduct business in English, not Spanish or Portuguese
	Reduced opportunities for researchers from indigenous communities (e.g. those from Quechua community in Peru vs. those from *mestizo* (“mixed race”) Peruvian community or of direct Spanish descent). As one Peruvian mentor expressed “Disparity is a relevant and important challenge in the Peruvian context. I literally know only three indigenous doctors”
	Failure of institutions to directly address the unconscious bias toward researchers from indigenous communities
	Issues of economic diversity are also important. As one mentor from Argentina expressed “Universities opening in poor areas may mean students may be the first person in their family to go to college. The important thing is for them to have a role model”
	Time difference between Latin America and the United States worked in faculty mentors’ favor, e.g. decisions could be made in real-time via email or phone call over the day
	The nomenclature of the word “mentor” was discussed at length with one investigator from Mexico stating “There is no word for mentor in Spanish”
	Mentorship should be defined—with all its varied and holistic facets—using terms in Spanish
	Government control over academic institutions means the government should be involved in changing the culture of mentoring and bringing mentoring as a focus to academia. As one investigator from Panama expressed, “in my country.. changes have depended on outside forces, by the Ministry of Science and Technology, for example. Perhaps to effect change in universities, we need to work with governmental governing bodies.”
	The importance of family and the interest of mentors in the mentee’s family life was raised in the life–work balance session with one participant stating “your career is nothing without family and we talk about that with our mentees”
Mombasa, Kenya, Workshop June 2013	“North–South issues” when grants or projects involve collaborations between Africa-based investigators and United States– or Europe-based investigators as delineated in the following text
	Collaborations not balanced. Investigators in the North make all the important decisions regarding funding and aims of the project
	Populations of interest to North-based global health researchers (e.g. those at risk for or living with HIV; individuals with malaria or TB) are in Africa, but the inclusion of Africa-based investigators on the project is perceived as “lip service” only from North-based investigators (e.g. to gain access to the populations of interest)
	Time difference was major detriment to the North–South collaborations. As one Africa-based investigator expressed “I wake up in the morning and my collaborators have all made important decisions over email in the middle of the night my time. How is that collaborative?”
	The post-colonial legacy in East African countries represented at the workshop contributed to this disparity in decision-making power and control of the research project’s trajectory. Specifically, Africa-based investigators stated that “we were told we were inferior to white people”. This internalized perception may influence interactions with North-based investigators in terms of acquiescence and giving their collaborators’ opinions more weight
	Mentees from the North can be given more time than locally based mentees, taking away from the time needed to build up local research capacity
	Similarly, visitors from the North on the investigative team are given prominence during their visit (e.g. in meeting with institutional leadership), even when they are more junior in academic rank. As expressed in one quote, “How come an Assistant Professor from xx University in the U.S. is given more time with the Dean than I have had in the last 20 years?”
	Salaries funded from grants from the NIH or Europe-based agencies for Africa-based faculty are much lower in absolute U.S. dollars than salaries funded in the North and should be higher
	African country investments in research were seen as important: “If the grant money comes from the U.S. and not from Kenya, the people from the U.S. get to dictate the terms”
	“Paternalism,” hierarchy, and respect for elders were also seen as barriers for honest, open mentor–mentee relationships. As one mentor expressed, “Where the mentor is considered the sun and should be worshipped.. mentees must be unassertive and worship”
	An emphasis on propriety rather than openness can lead to “authority and value being given to a bad mentor instead of telling him the truth”
Bangalore, India, workshop November 2014	Issues of hierarchy were predominant in the discussions at the South Asia–based workshop regarding the mentee–mentor relationship
	A mentee-driven process, as encouraged by the didactic presentations, was seen as difficult in the South Asia context as mentees are supposed to defer to their professors’ needs and opinions
	The nomenclature of “supervisor” vs. “mentor” was discussed at length in this workshop because supervisors for a mentee’s research project are often assigned by the institution without consideration of the potential for true mentorship (e.g. aiding in the mentee’s success; taking the mentee’s research interests into account; aiding in the visibility of the mentee by introducing her/him to collaborators in the field; taking an interest in the mentee’s life–work balance
	True bidirectional feedback in the context of a hierarchical system is not usually encouraged. As expressed by one mentor from Bangladesh, “I don’t think my mentee would ever really tell me if he was unhappy with my mentoring”
	Issues of caste and economic class also raised as barriers to mentoring and barriers to the success of early-stage investigators from lower economic strata
	Unconscious bias toward investigators from traditionally lower castes is not often addressed in the South Asia setting
	Post-colonial legacy can lead to deference to North-based investigators, although this point was not raised as frequently as in the Kenya-based workshop
	Gender dynamics raised frequently with female investigators citing bias toward them when they start a family with the automatic assumption that they will no longer work as productively. As expressed by a senior female investigator in Bangalore, “As soon as I had my first child, my colleagues were asking me if I was going to take more time off or ask for a leave”
Johannesburg, South Africa, workshop March 2016	Issues of the post-apartheid legacy and its continued effects were predominant in the discussions regarding mentoring and mentoring effectiveness in South Africa
	Deans, department chairs, and section heads in South Africa–based institutions tend to be White
	It is more difficult for Black investigators (or those of mixed race or Indian descent) to rise in academic rank
	Issues of race, disparity, and both conscious and unconscious bias affect both faculty morale and the mentor–mentee interaction. As expressed by one faculty mentor from the Xhosa ethnic group in South Africa, “I cannot even get my Dean to pronounce my name properly, let alone recognize me for promotion”
	Although unconscious bias may be addressed and talked about in the South African context, those discussions do not always lead to changes in the biased nature of the system
	Zimbabwe-based investigators expressed that the expulsion of Whites under the former Mugabe administration was detrimental to the academic enterprise and to long-standing collaborations, although admittedly that bias in academia had also led to Whites being granted positions of leadership
	Resources were more available for mentoring training and structured mentoring programs in South Africa, but needed to be harnessed for greater efficacy
	Gender dynamics were discussed in the context of senior male faculty members having impunity from “power imbalances with female mentees”

NIH = National Institutes of Health.

Finally, Figure 1 shows an example of major themes that emerged in the Mentoring Action Plans from the Mentoring the Mentors workshop in South Asia in November 2014, illustrating both the challenge and possible solutions to mentoring in the region.

### April 2017 technical advisory meeting.

During the fifth year of the program and after the final workshop described here, GloCal hosted a senior-level technical advisory meeting on global health mentoring focused on LMIC institutions in Virginia in April 2017. With representation from the leadership of all six consortia, the NIH FIC, and LMIC faculty leaders from the four “Mentoring the Mentors” workshops, the success of the workshops in launching local mentoring initiatives were discussed. At many of the participating institutions, deans and department chairs had committed to establishing mentoring programs, although many of them were still nascent. The group discussed the need for ongoing FIC presence in LMICs to encourage the development of local mentoring capacity and structured programs.

The 47 respondents to the pre-meeting surveys were from 19 LMICs (46% African, 39% South Asian, and 14% Latin American). They reported medians of 18 years of experience in research and 10 years in mentoring trainees. Nineteen were institutional leaders (deans, directors, and department chairs). Only 16 of the 47 respondents had attended one of the four regional workshops. Of these respondents, 11 had shared their mentorship training experience with colleagues in their institutions, six had been able to set up mentorship training workshops or other training opportunities in mentoring within their institutions, and 11 had had opportunities to meet with other mid-level and senior mentors to discuss mentoring.

All surveyed individuals were asked whether their institutions have any mentorship programs; 23 of 40 respondents (57.5%) indicated that their institutions did, with 19 of the 23 programs focused on mentees and four geared toward mentor training. Only 11 of the programs (48%) included any evaluation components. There was general support for, and ideas offered, on the possibility of regional mentor training programs to assist institutions that may not be able to launch them on their own. Factors considered key to successful regional programs included clear support from one’s own institution, especially with regard to sustainability, and funding.

The survey ascertained whether there was a hierarchical relationship between mentor and mentee at the respondents’ institutions, and how much they thought a hierarchical relationship prohibits effective mentoring. Both questions offered Likert scales where 1 = not hierarchical or no impact, and 10 = extremely hierarchical and high impact. Median responses to both questions were five, including both presence and impact of hierarchical cultures. The final question asked whether mentor training programs could reduce the institution’s hierarchical mentoring culture. Of 34 responses, 19 (55%) said yes, 14 (42%) said maybe or not sure, and only one (3%) said no, implying some degree of optimism for change.

In free-text comments entered about challenges and barriers respondents faced in institutionalizing mentorship training, most cited competing time commitments or simply insufficient time. Some individuals also cited lack of sufficient numbers of trained mentors and of mentorship training programs, and lack of prioritization of mentor training. Additional barriers included siloing or lack of communication/coordination among specialties and research groups, and lack of institutional mentorship cultures or mandates from institutional leaders. By contrast, however, several respondents indicated that their leaders were quite receptive to instituting mentorship training. Additional challenges included lack of funding for mentorship training and lack of follow-up training to sustain mentorship skills.

Success stories launched by the NIH FIC mentoring initiatives were shared by 10 workshop attendees, including growth of institutional support and establishment of several new institutional mentorship training programs, and initiation of peer mentorship networks, regular mentor–mentee meetings, and IDPs. One newly established mentorship training model in Latin America was reported as being expanded as a national mandate for research training, nested within a required training program in the responsible conduct of research.

## Conclusion

This article describes robust regional “Mentoring the Mentors” workshops designed to train mid-level and senior faculty in LMICs on techniques and tools to improve mentoring practices for early career investigators and other trainees. The four workshops were conducted from 2013 until 2016 in Lima, Peru; Mombasa, Kenya; Bangalore, India; and Johannesburg, South Africa, and trained mostly mid-level and senior faculty, including department chairs and deans, actively mentoring trainees in global health research from surrounding countries. Each program was co-led and tailored by local experts and faculty leaders from each host institution and surrounding institutions who facilitated each workshop in collaboration with faculty leaders from the United States—based FIC consortia.

There were a number of shared barriers to mentoring across all institutions, including a lack of a culture of mentoring, time constraints , lack of formal training and a lack of recognition (in terms of either remuneration or advancement in academic rank) for mentoring. A number of creative and robust solutions to the barriers associated with mentoring at LMICs were proposed by the 132 participants in the four workshops, focusing at both the institutional and individual levels. The most consistent solution proposed to effective mentoring was the establishment of sustainable mentoring training programs such as the ones described in this article, dissemination of the training materials associated with these workshops, and the formalization and recognition of the mentoring process within academic institutions to inculcate a culture of mentoring. In the context of newly developed mentorship programs, formal and well-structured evaluation of such programs will be necessary.^23^ Moreover, the discussion in the four workshops suggested a series of mentoring tools that can be applied and standardized across institutions.^24^

A number of contextual barriers to mentoring were raised in the four workshops. Although these contextual barriers suggest a number of solutions that can only be enacted locally with support from institutional leadership, these barriers require global health researchers from high-income countries to be sensitive to the local context, and ensure equitable partnerships based on mutual respect and collegiality. Finally, a post-workshop survey administered to both workshop attendees and faculty leadership at LMIC institutions associated with the FIC training programs offered hope and optimism in growing both a mentoring culture and culturally specific mentoring programs to enhance the success of global health researchers and advance health around the world.
